# Bovine pericardium retail preserved in glutaraldehyde and used as a vascular patch

**DOI:** 10.1186/1471-2482-11-37

**Published:** 2011-12-22

**Authors:** Wladimir F Saporito, Adílson C Pires, Sérgio H Cardoso, João A Correa, Luiz Carlos de Abreu, Vitor E Valenti, Luciano MR Miller, Eduardo Colombari

**Affiliations:** 1 Departamento de Cirurgia and Laboratório de Escrita Científica, Departamento de Morfologia e Fisiologia, Faculdade de Medicina do ABC, Santo André, SP, Brasil; 2 Laboratório de Escrita Científica, Departamento de Morfologia e Fisiologia, Faculdade de Medicina do ABC, Santo André, SP, Brasil; 3 Departamento de Fonoaudiologia, Faculdade de Filosofia e Ciências, Universidade Estadual Paulista, UNESP, Marília, SP, Brasil

## Abstract

**Background:**

In this study we evaluated the performance of bovine pericardium preserved in glutaraldehyde used as a vascular patch.

**Methods:**

Fourteen young pigs, six females and eight males, weighting 10.3 - 18.4 kg were used in our study. We implanted three remnants in each pig, two in the abdominal aorta and one was juxtaposed to the peritoneum. The smooth face (SF) and rough face (RF) of each remnant were implanted turned to the vessel inner portion and one remnant was juxtaposed to the peritoneum. The animals were sacrificed in 4.5 - 8 months after surgery (75 - 109 kg). The remnants were assessed for aorta wall, fibroses formation in inner apposition and calcification related to the face turned to the vessel inner portion.

**Results:**

The rough face showed a lower dilatation level compared to the face implanted in adjacent aorta. There was no difference between intensity and/or incidence of graft calcification when the superficies were compared. The bovine pericardium preserved in glutaraldehyde did not show alterations in its structure when implanted with different faces turned to the inner portion of vessel.

**Conclusion:**

When turned to the inner portion of the vessel, the rough face of the remnant presented a lower dilatation in relation to the adjacent aorta and a better quality of endothelium layer and did not show a difference between intensity and/or incidence of graft calcification.

## Background

The use of biological tissues such as vascular substitutes dates back to early days of cardiovascular surgery, when an autologous vein was used as an arterial substitute. The use of prostheses and orthoses have become necessary to the development of cardiovascular surgery, since the correction of complex congenital heart disease and valvular heart disease require the use of these materials as vascular substitutes [1]. Carpentier et al [2], with the introduction of glutaraldehyde at low concentration for the preservation of biological tissues, started a new period by introducing the concept of bioprosthesis, which despite being a biological tissue, it loses its antigenicity, does not induce the formation of antibodies and produces the disruption of intra and intermolecular protein, enhancing its structural stability, decreasing its antigenicity and maintaining the sterile tissue.

The pericardium treated with glutaraldehyde is one of the biological materials more widely used in cardiovascular surgery [3-5]. Despite the good results obtained in clinical trials, degeneration of biological tissue is frequently observed, especially calcification, which is the major cause of dysfunction of these tissues that may cause mechanical failure and degeneration [6-8].

Pires et al [9,10] described the profile of bovine pericardium preserved in glutaraldehyde, while employed as a vascular patch. They reported large functional differences between the smooth (membranous) and rough (fibrosis) face of the pericardium. These differences depended on the location. The authors suggested a possible correlation between calcification and the contact of the rough face of the pericardium with the bloodstream. Several experiments were conducted in order to avoid or reduce the calcification of glutaraldehyde-treated pericardium [9,10], but none was able to completely inhibit the calcification of the material in question.

We tried to understand the mechanisms of this dysfunction, which is not fully elucidated yet. One aspect to consider is that the bovine pericardium presents two distinct sides, the smooth and rugged faces [11]. In clinical practice the smooth side is usually used with the smooth side facing the lumen [12]. However, no studies have described the differences between the two sides (smooth face vs. rough face). Therefore, we aimed to evaluate the performance of bovine pericardium preserved in glutaraldehyde used as vascular patch and we analyzed the possible differences between smooth and rough faces in contact with the bloodstream.

## Methods

We used commercially produced bovine pericardium. The material used in our study was produced by Braile Biomédica^® ^industry. The remnant was made by using a metal elliptical mold, measuring 2.0 cm in the greatest diameter and 1.0 cm in the smallest diameter. The surgical procedure was performed under sterile conditions in the operating room of the department of surgical technique of our Institution. This study was approved by the Ethics Committee in Research on Animal Experiments of our Institution (Number 1808/99).

**Figure 1:**
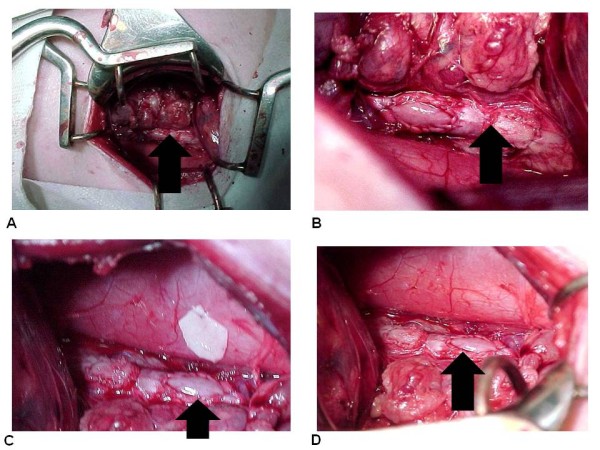
**(A) Overview of the surgical field**. (B) Retail is implanted in the abdominal aorta. Immediate aspect. (C) Bovine pericardium patch implanted in the aorta and peritoneum. (D) Overview of bovine pericardium implanted in the aorta. Final aspect. Arrows indicate the position of the implants.

We implanted the standardized retails of bovine pericardium preserved in glutaraldehyde, obtained from the Gravataí industry in 14 young Large White breed pigs, weighing between 10.3 kg and 18.4 kg, six females and eight males, We implanted three remnants in each pig, two in the abdominal aorta and one was juxtaposed to peritoneum, with a smooth face (SF), the other with the rough face (RF) facing the lumen and one remnant was juxtaposed to peritoneum (Figure 1). We followed the following standardized procedures:

Animals were kept in the immediate postoperative period in the vivarium of our University, where they received antibiotic prophylaxis with intramuscular penicillin G benzathine (Sigma^®^) at a dose of 600,000 units, analgesia with tenoxicam (Sigma^®^) in the first 24 hours after surgery, and all the necessary postoperative care including feeding, vaccinations and other medications, supervised by a veterinarian. They remained on average until the seventh postoperative day, depending on their clinical conditions. They were then transferred to a farm specializing in raising pigs where they received usual care as needed.

The animals were divided into three groups according to their postoperative period, ranging from 4.5 to 8 months, and weighing between 75 to 109 kg. They were sacrificed using a lethal dose of sodium pentobarbital (Sigma^®^). Segments of the aorta with the location of the implants were removed and longitudinally opened opposite to the implant. The aorta segment which was opened was fixed on a cork board in order to keep its shape, with the inner side facing down and fixed in aqueous 10% formaldehyde (Sigma^®^). We evaluated the deformation of the retail which is characterized by its expansion in relation to the adjacent aorta.

The removed parts were analyzed at the Department of Pathology of the Universidade Federal de São Paulo. The pieces were cut along the longest axis of the retail and embedded in paraffin. Fifteen sections (8-15 μm) were cut from each tissue sample. Slides were stained by the hematoxylin - eosin method and the method of Verhoeff.

The removed parts were macroscopically and microscopically evaluated. We used criteria from previous studies to conduct a quantitative analysis of each slide as follows [13-15]:

Calcification: characterized by deposition of calcium in the intimacy of the retail and the adjacent aorta. The presence of one to two foci was considered to be mild calcification (1st degree), three foci were considered moderate (2nd degree), and more than three foci or confluent areas of calcification were considered intense (3rd degree).

Integration tissue: composed of fibrous connective tissue responsible for the integration of the bovine pericardium retail to the aorta.

Internal apposition fibrosis: characterized by the presence of fibrous tissue in the face of the pericardium toward the interior of the aorta. Internal apposition fibrosis was assesses according to the extent and thickness. The extent was considered mild (1st degree) when covering less than half of the retail area, moderate (2nd degree) when covering half or more of the retail area and intense (3rd degree) when covering the entire area of the retail. The thickness was considered mild (1st degree) when its thickness was lower than the retail thickness, moderate (2nd degree) when its thickness was equivalent to the retail thickness and intense (3rd degree) when its thickness exceeded the retail thickness.

Bone metaplasia - cartilaginous: characterized by the formation of bone tissue - cartilage in the fibrous layer of the internal apposition. It was considered mild (1st degree) in the presence of one to two foci, moderate (2nd degree) in the presence of three foci, and intense (3rd degree) in the presence of more than three foci or confluent areas.

The experiments lasted a total of 14 months and the surgical procedures were performed by the same surgical team. Data were reviewed by two independent readers.

In order to evaluate the association between quantitative variables, we applied the chi-square or Fisher exact test. Differences were considered significant when the probability of a Type I error was less than 5% (p < 0.05).

## Results

**Figure 2:**
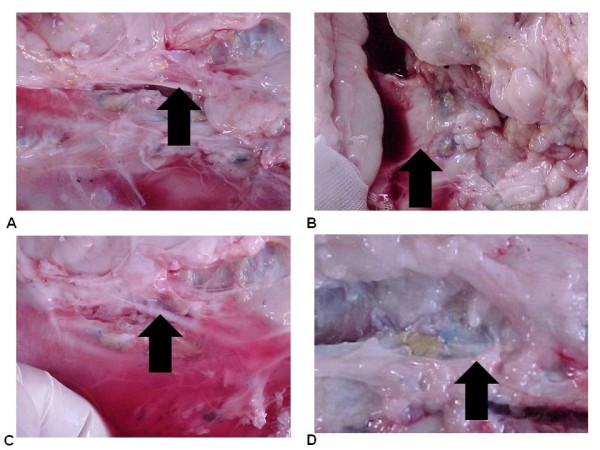
**(A) Aspect of the patch at the postoperative period (animal 3)**. (B) Aspect of the patch at the postoperative period (animal 7). (C) Aspect of the patch at the postoperative period (animal 11). (D) Retail integrated to the aorta. Arrows indicate the position of the implants.

All animals progressed well in the immediate and late postoperative period, all stayed asymptomatic with good growth and adequate weight for their age; we did not observe any deaths. Only one of 14 animals (7.14%) developed wound infection, requiring prolonged hospitalization in a vivarium, treated with antibiotics. The patches were well incorporated into the aorta of the animals, keeping their limits, irrespective of the side that was facing the lumen (Figure 2).

We observed dilation of the surgical patch area in relation to the surrounding aorta in both groups. In the SF group dilation occurred in all animals and was mild in almost half. On the other hand, in the RF group we did not observe dilation in most animals (Table 1).

**Table 1:** Evaluation of the aortic patch and the expansion of the retail implanted in the aorta in relation to the adjacent aorta.

	*SF*	*RF*	*p*
***Zero***	-	12	< 0.05
***1^st ^degree***	6	2	< 0.05
***2^nd ^degree***	4	-	< 0.05
***3^rd ^degree***	4	-	< 0.05

Statistical test: Fischer's test. RF: Rough Face - Retail dilatation; SF: Smooth Face - Retail dilatation. (Zero) absent; (1^st ^degree) light, when reaching a depth of 1.0 mm in its longest axis in relation to the adjacent aorta; (2^nd ^degree) moderate, when reaching a depth of 2.0 mm in its longest axis in relation to the adjacent aorta; (3^rd ^degree) intense, when reaching a depth of 3.0 mm or more in its longest axis, in relation to the adjacent aorta.

The calcification of the surgical patch occurred in both groups. In the SF group it was absent in more than a third of the animals, while in the RF group it was absent in over half of the animals (Table 2). The calcification of the aorta adjacent to the vascular patch was observed in nine animals in the SF group (Table 3) and in six animals in the RF group (Table 4). We did not observe calcification in the retail implanted in the peritoneum in approximately 40% of the animals (Tables 3 and 4).

**Table 2:** Evaluation of the aortic patch in relation to the calcification of the retail implanted in the aorta.

	*SF*	*RF*	*P*
***Zero***	5	8	0.1
***1^st ^degree***	4	5	0.23
***2^nd ^degree***	3	1	0.12
***3^rd ^degree***	2	-	0.4

Statistical test: Fischer's test. SF: Smooth Face - Retail calcification; RF: Rough Face - Retail calcification. (1^st ^degree) up to 2 small foci; (2^nd ^degree) up to 3 small foci; (3^rd ^degree) more than 3 foci.

**Table 3:** Evaluation of the peritoneal retail in relation to calcification compared to the retail implanted in the aorta with smooth face.

	*PR*	*SF*	*p*
***Zero***	6	5	0.34
***1^st ^degree***	3	4	0.2
***2^nd ^degree***	5	3	0.14
***3^rd ^degree***	-	2	0.6

Statistical test: Fischer's test. PR: Peritoneal Retail - Calcification of the peritoneal retail; SF: Smooth Face - Calcification of the retail. (1^st ^degree) up to 2 small foci; (2^nd ^degree) up to 3 small foci; (3^rd ^degree) more than 3 foci.

**Table 4:** Evaluation of the peritoneal retail in relation to calcification compared to the retail implanted in the aorta with rough face.

	*PR*	*RF*	*p*
***Zero***	6	8	0.31
***1^st ^degree***	3	5	0.54
***2^nd ^degree***	5	1	0.47
***3^rd ^degree***	-	-	0.57

Statistical test: Fischer's test. PR: Peritoneal Retail - Calcification of the peritoneal retail; RF: Rough Face - Calcification of the retail. (1^st ^degree) up to 2 small foci; (2^nd ^degree) up to 3 small foci; (3^rd ^degree) more than 3 foci. Evaluation of peritoneal retail regarding calcification, compared to the retail implanted in the aorta with the Rough Face faced to the light.

The coaptation between the bovine pericardial patch and the aortic wall was noted by apposition or slight invagination of edges in 12 cases. In two cases one side presented a slight overlap the edges. The integration between the retail and the aortic wall was observed in a thin band of fibrous connective tissue that also involved the suture.

In the group in which the flat edge of the retail was turned into the aorta, this phenomenon was more intense on the suture line. In the RF group the extent of coverage by internal apposition fibrosis of the retail area was moderate (2nd degree) in most animals (Table 5). In the group in which the flat edge of the retail was turned into the aorta, this phenomenon was more intense on the suture line and it occurred in both groups. In the SF group the thickness of the internal apposition fibrosis in relation to the retail area was slight (1st degree) in most animals while in the RF group we found intense thickness in all patches (Table 6).

**Table 5:** Evaluation of aortic patch in relation to the coverage area of internal apposition fibrosis of the retail deployed in the aorta.

	*SF*	*RF*	*p*
***Zero***	-	-	< 0.001
***1^st ^degree***	11	-	< 0.001
***2^nd ^degree***	3	10	< 0.001
***3^rd ^degree***	-	4	< 0.001

Statistical test: Chi Square test. SF: Smooth Face - Extension of fibrosis; RF: Rough Face - Extension of fibrosis. (Zero) Absent; (1^st ^degree) light, when covering less than half of the retail area; (2^nd ^degree) moderate, when covering more than a half of the retail area.; (3^rd ^degree) intense, when covering the entire area of the retail.

**Table 6:** Evaluation of aortic patch in relation to the thickness of the internal apposition fibrosis of the retail deployed in the aorta.

	*SF*	*RF*	*p*
***Zero*** ***1^st ^degree***	- 9	- -	< 0.001 < 0.001
***2^nd ^degree***	4	-	< 0.001
***3^rd ^degree***	1	14	< 0.001

p < 0.001 - Chi Square test. SF: Smooth Face - Thickness of fibrosis; RF: Rough Face - Thickness of fibrosis. (Zero) Absent; (1^st ^degree) light, when the fibrosis thickness was lower than the retail; (2^nd ^degree) moderate, when the fibrosis thickness was the same of the retail; (3^rd ^degree) intense, when the fibrosis thickness was higher than the retail.

**Figure 3:**
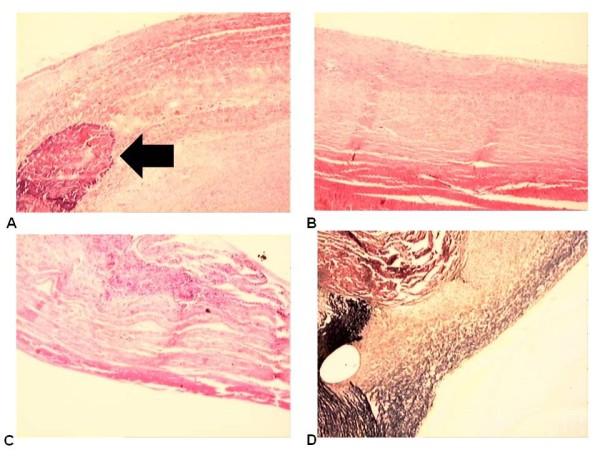
**(A) Aspect of the structure of the patch with calcification (hematoxylin - eosin - 4×)**. (B) Aspect of the cover layer on the inner face of the patch (RF) (hematoxylin- eosin- 4×). (C) Aspect of the cover layer on the inner face of the patch (SF) (hematoxylin - eosin - 4×). (D) Aspect of the cover layer on the inner face of the patch (RF) (Verhoeff - 4×). Arrows indicate calcification.

In both groups we found a fibrous layer internal apposition that consisted of dense fibrous tissue predominantly modeled, and the cells and fibromas were compactly arranged parallel to each other, acquiring the pattern of tendons and ligaments tissue (Figure 3).

There was only one animal (7.1%) in the RF group that presented osteo-cartilaginous metaplasia in the internal apposition fibrous layer.

The anatomic and pathologic evaluation showed that all parts of the bovine pericardium were conserved. Moreover, we found a superposition of a fibroelastic dense connective tissue collagen fibers on the face found facing the lumen of the vessel, which was modeled in a compact arrangement and arranged parallel to the surface, similar to modeled dense connective tissue. There were no degenerative, inflammatory or metaplastic diseases.

## Discussion

Our main finding was that the bovine pericardium preserved in glutaraldehyde presented no changes in its structure when deployed with different faces to the vessel lumen. Its production followed a rough standardization of manufacturing and quality control. In order to reduce the variables that could influence the results, the pericardium was obtained from only one source and the patch was made with the aid of the same mold.

It is important to note the presence of an expansion of the retail in the aortic wall. There was dilation of the material with the formation of an aneurysm wall at the implant site, which was significantly higher when the smooth face was turned to the light of the aorta. Furthermore, we observed that the bovine pericardium patch was well integrated into the wall of the aorta, confirming what Pires et al [9] observed. The structure of the patch provided by surgical retail, when implanted in the aorta of dogs, promotes clear and precise limits in preserved shape, and it is already well defined at the end of the first month after surgery [9,10]. Also, this type of procedure is vulnerable to infections [16-18].

In the group where the rough face was turned into the aorta, we observed the formation of a covering layer on the inner surface with more plasticity, modeling elastic tendon and bone formation, which provided greater tension resistance to the surgical patch, preventing its expansion. Pires et al [9,10] suggested that the internal apposition fibrosis originates from the fibrous organization of blood components deposited on the surface of the retail, because this plan was not observed in any case in retail implanted in the pericardium. In the group in which the flat edge of the retail was turned into the aorta this phenomenon was more intense on the suture line and was related to the roughness of the area and local release of thromboplastin by damaged tissues. Our results suggest that the wrinkled face of the retail would facilitate the capture and adhesion of blood components across its surface, with subsequent assemblage by the release of platelet factors and secondarily by thromboplastin released from damaged tissues [19]. We also verified that in this group there was the formation of a thin inner cover layer, resulting in dilatation of the patch area of surgery. These results may suggest that fibrosis internal apposition would support retail and strengthen the structure of the surgical patch. Its histological structure reveals a dense fibrous connective tissue shaped pattern tendon with great tensile resistance.

Researchers have offered different hypotheses to explain why glutaraldehyde helped preserve bovine pericardium from changes in its structure when deployed with different faces to the vessel lumen. Ishihara et al [11] reported that bovine pericardium treated with glutaraldehyde loses the mesothelial cells of serosal surface, exposing the submesotelial layer of connective smooth tissue, while Pires et al [9] observed that the surface of the pericardium was covered by a layer of pavement cells and fibrous connective tissue, which they called internal apposition tissue. On the other hand Schoen et al [8] reported that platelet aggregation, on the pericardial membrane, would be a factor for accelerating the calcification process. We observed that in both sides of the retail implanted in animals, an inner surface composed of endothelium was formed. However, when the retail was implanted with the rough side facing the lumen surface, it was isolated from bovine pericardium through blood, thus reducing the intensity and incidence of aortic dilatation in relation to the adjacent aorta. Without this coverage, we would expect an increase in calcification. Additionally, we observed that this layer of tissue was differentiated from the other sides and better trained when it had the wrinkled face toward the lumen. Furthermore, modeling elastic tendon and bone formation, formed a surgical patch level with the retail bovine pericardium which had good resistance and was well integrated into the aortic wall, probably because of the uniformity resulting from the molding process.

The presence of osteo-cartilaginous metaplasia in the fibrous layer of internal apposition was observed in only one case as an isolated focus, occurring in the group in which the rough face was turned into the aorta. These data are not in agreement with the findings of Pires et al [9] who described a large amount of calcium present in the inner layer of apposition. The authors hypothesized that this calcification represented an osteo-cartilaginous metaplasia and ossification of fibrous tissue covering the graft, indicating the transformation of fibroblast into osteoblasts. Nonetheless, we feel confident that our findings are valid. Our methodology was validated by Sucu et al [20] who demonstrated that the use of microscopic sections merged for evaluation of calcification was more precise than the use of the chemical method of extraction of calcium from the patch.

The calcification of biological tissue is the main cause of bioprostheses dysfunction. According to Schoen et al [7,8], Chanda et al [21], Vasudev et al [22] and Pires et al [9] this calcification is a multifactorial phenomenon, but it is not well defined and varies in different animal models. The authors pointed out that calcification in bovine pericardium used as a vascular patch presented a different connotation of calcification than valvular prostheses, because it implied a reduction in leaflet mobility, resulting in dysfunction of the prosthesis, which was not observed in vascular grafts.

We observed calcification of the retail irrespective of the side facing the lumen; the same was true regarding the retail implanted in the peritoneum of the animal. However, these finding are not consistent with findings of Schoen et al [6] and Rossi et al [23] who found calcification of retail implanted in rats. The authors described calcification of bovine pericardium from 24 hours of implantation and progressively increased with time, but did not report the presence of internal tissue apposition. We did not find this tissue in the patches implanted in the peritoneum (without direct contact with the bloodstream). These findings are consistent with findings by Gabbay et al [24], Bortolotti et al [25] and Pires et al [10], who indicated the major influences of the implant site of bovine pericardium treated with glutaraldehyde and its direct contact with the bloodstream.

We used a bovine pericardium produced by Braile Biomedica. However, several other industries also produce the same patch. Some industries use special procedures to reduce the prevalence of calcification. For example, Synovis, who produces Vascu-Guard [26], follows Apex-Processing. Briefly, in this process, the levels of residual glutaraldehyde are below the limits of detection by the sophisticated analytical methods now available (< 0.5 ppm) and products undergoing Apex-Processing have levels of cellularity that are four times lower than a variety of competitive materials including products conventionally treated.

According to our data, there was no calcification in the retail implanted in the peritoneum in almost half of the animals. Peritoneal calcification is a rare condition developed in uremic patients on continuous ambulatory peritoneal dialysis. Once peritoneal calcification is detected, it is essential to assess whether encapsulating peritoneal sclerosis develops [27].

Bovine pericardium is also implanted for the vascular reconstruction in the femoral or carotid artery as patchplasty and venous patch angioplasty [28]. Moreover, the bovine pericardium is not only used in cardiovascular surgery but also in hernia [29] or thoracic surgery [30]. Based on our data, we confirm the use of the rough face in those types of surgery. We propose future studies to investigate this possibility.

Our investigation presents some points that should be addressed: we did not perform blood analysis, i.e., cholesterol, triglycerides and blood glucose levels. However, we aimed to focus only on calcification. Hematoxylin and eosin is not the best method for investigating tissue calcification and it does not quantify proteins related to fibrosis. On the other hand, this is the first study to investigate the utilization of different faces of bovine pericardium conserved in glutaraldehyde as a vascular patch. The glutaraldehyde treatment, the resulting increased stiffness of the treated tissue and the presence of fixed cellular material in the tissue are all factors contributing to the calcification of cardiovascular implants. We did not perform microbiological investigation of the patch. We suggest this procedure for future studies. The time the bovine patch stayed in the circulation was not constant, possibly this factor may be a bias for our findings.

## Conclusion

When turned to the inner portion of the vessel, the rough face of the remnant had a lower dilatation in relation to the adjacent aorta and a better quality of endothelium layer and there was no difference between intensity and/or incidence of graft calcification. Therefore, we suggest the surgeons to use the smooth face.

## Competing interests

The authors declare that they have no competing interests.

## Authors' contributions

All authors participated in the acquisition of data and revision of the manuscript. All authors determined the design, performed the statistical analysis, interpreted the data and drafted the manuscript. All authors read and gave final approval for the version submitted for publication.

## Pre-publication history

The pre-publication history for this paper can be accessed here:

http://www.biomedcentral.com/1471-2482/11/37/prepub
